# Supplementary material for: Chromothripsis during telomere crisis is independent of NHEJ, and consistent with a replicative origin
Source: Genome Res. 2019 May;29(5):737–49. doi: 10.1101/gr.240705.118 (PMC6499312; doi:10.1101/gr.240705.118)
Supplement: Supplemental Material [file supp_gr.240705.118_Supplemental_file_1.zip › contigs/annotated_contigs/DB90/contig.2.DB90_length_585_mean_cov_15.3846153846.docx]

**DB90_length_585_mean_cov_15.3846153846**

```
ACTTGTTACCCACATTCATGTAGATAATAGCTCCAGCTTTGGCTAATATGTCCCTCCCTAATAAGGGTGTGAGACTTTCAGGCATAATG
 >chrX:111356514-111356883 + E=2e-210
AGAAAGGCATGTGAAAAGAGCAAAGTCTCCCAATTACAACTGAGGAGGTGGGAGAAATACCTGGTTACAGCTATCCCAGGATTCCTTGG

ATGGTAACGGACTTTGAGGACAGCTGTCCCGGACAGGAGAGTAACACTGAGAAAGCCATGCCAGTGTCCAGGGGGAAGTCAATTTCCTA

GCCCTCAATGGTTAAATGTACCCAGGGCCCAGTGAGGGTGGTGACATGAGCTGGCGCTTGCTCCGGGCACCCTCAGTCCTATTGTTGAA

TCATCTGGTTGT|G|AGCGAATATTGGGGCCAGGCCATAGTGCAGAAAAAAATAAGCTGCTTCTTTTTCAGGGTTTTTGGGTCAAATTG
             >chrX:111358178-111358395 + E=7e-119
GTCCCAATGGCATAGGATGCATTTCAAGGGTGAGCCTGTTGATGCCTGAGTGTTTCCCATCTGAAAGAAAAAACTGCCTGCGGTTCTGA

TTTGTTTTTCCCCCACCACCCAAGAACCCACAACAGTCTTTGGACCCTGCTCA
```
